# Evaluation of Physical Symptoms in Patients on Peritoneal Dialysis

**DOI:** 10.1155/2012/305424

**Published:** 2012-09-25

**Authors:** Ana Elizabeth Figueiredo, Cate Goodlad, Michelle Clemenger, San San Haddoub, Jacqueline McGrory, Kim Pryde, Emma Tonkins, Nora Hisole, Edwina Anne Brown

**Affiliations:** ^1^Faculdade de Enfermagem, Nutrição e Fisioterapia, Pontifícia Universidade Católica do Rio Grande do Sul, FAENFI/PUCRS, 90619.900 Porto Alegre, RS, Brazil; ^2^West London Renal and Transplant Centre, Hammersmith Hospital, London W12 OHS, UK

## Abstract

*Introduction*. Little is known about physical symptoms in peritoneal dialysis (PD) Patients. This study aims to determine the prevalence of symptoms (general and abdominal) in PD patients. *Methods*. A cross-sectional study, with subsequent followup, using an author-designed 21 symptoms questionnaire (15 nonabdominal and 6 abdominal). Each symptom was assessed on a scale 0–3 for severity (none–severe) and frequency (never–every day). *Results*. We studied 41 patients, mean age 60 ± 15 years, 56% male, 19.5% diabetics, and 51.5% on APD. Mean number of symptoms was 9.5 ± 3.9 and total symptoms score was 28.5 ± 12 with abdominal scores of 6.4 ± 4.8. Most frequent symptoms were lack of energy, itching, cramps, poor sleep, and loss of appetite. A second evaluation in 20 patients disclosed no statistical difference between the first and second assessments, or between subgroups. Cramps were the only symptoms which decreased over time (*P* = 0.120). Lack of energy did not correlate with haemoglobin, neither did itching with phosphate level. *Conclusions*. Physical symptoms are frequent and troublesome; they relate to advanced kidney disease and not specifically to PD. Symptoms remain stable over time and do not appear to relate to dialysis parameter markers.

## 1. Introduction

Peritoneal dialysis is a life-sustaining treatment for patients with end-stage renal disease–a chronic illness in which physical and emotional symptoms play a central role in patient's experience. Symptom burden among dialysis patients can be underdiagnosed and undertreated by providers of care, and the frequency and severity of many symptoms underestimated [1, 2].

Specific symptoms questionnaires, such as the Dialysis Symptom Index [3], the Edmond Symptom Assessment System [4], and the Dialysis Frequency, Severity and Symptom Burden Index (DFSSBI) [5] have been developed, but have been designed predominantly for patients on hemodialysis. Reported quality of life for dialysis patients is lower than in the general population, and it has been suggested that this is a result of comorbidities, lifestyle adaptations, and treatment-related side effects [6–8]. 

The BOLDE study [9] has previously reported that older patients on PD have less illness intrusion compared to those on HD, and that symptom count is a significant negative contributor to quality of life.

The current literature provides an incomplete picture of the prevalence; severity and clinical significance of the symptoms burden in peritoneal dialysis patients, and the main focus has been on single symptoms rather than a range of symptoms.

Symptoms represent patients experience so it is not surprising that patients identify symptom management as very high priority [10]. Knowledge and comprehension of the prevalence of symptoms in peritoneal dialysis patients can therefore guide the development of medical and nursing interventions and practice to be specifically targeted at symptoms. Therefore, the purpose of this study is to determine which symptoms are mostly reported in peritoneal dialysis patients using a modified Dialysis Symptom Index.

## 2. Patients and Methods

This is a cross-sectional study in which 41 patients with more than three months on PD completed a symptom survey, of those 20 subsequently completed the survey a second time. Demographic and clinical data were collected from patient records and during the study assessment. The most recent routine blood test results, total weekly creatinine clearance, and PET values were recorded. All peritoneal dialysis patients were invited to take part. Patients who were not able to read and understand English or the ones with severe visual deficiency were excluded. As existing symptom assessments have been developed for HD, symptoms were evaluated in this study using a modified questionnaire designed by the authors to include symptoms relevant to PD without making the questionnaire too long.

 A total of 21 symptoms were evaluated. There were 15 questions relating to nonabdominal (joint pain, blurred vision, weakness, headache, dizziness, cold hands, cramps, shortness of breath, unsteadiness, dry mouth, poor sleep, edema, itching, lack of energy, and nausea) and 6 questions relating to abdominal symptoms (vomiting, abdominal pain, abdominal distention, constipation, diarrhea, and loss of appetite). Summing the number of symptoms reported generated an overall symptom burden score ranging from 0 to 21. Each symptom would receive 0 to 3 points related to severity according to the following scale: none (0), mild (1), moderate (2), or severe (3). Scores from 0 to 3 were also attributed to frequency as never (0), once a week or less (1), most days but not all (2), and every day (3). Therefore an overall symptom (severity/frequency) score ranging from 0 to 126 (90 for general and 36 for abdominal symptoms) was generated. The questionnaire was self-completed by the patients during their clinic visits as part of their usual clinical care and kept with the patients records. Alternatively, patients were allowed to complete the questionnaire at home and send it back by post. The study was performed at the PD Unit of Hammersmith Hospital; data collection was from 2008 to February 2011.

Data are presented as means and standard deviation (SD) or medians and interquartile range (IQR) and frequency and percentages. Normality of variables was evaluated by the Kolmogorov Smirnov test. Comparisons between the two surveys were performed using Paired Student *t*-test or with Wilcoxon-Mann-Whitney tests. All statistical analyses were undertaken using SPSS 17.0 with a significance level (alpha) of 5%. Pearson or Spearman Correlation Coefficient (*r*) was used to assess the relationship between symptoms and clinical parameters.

## 3. Results

Demographic and clinical characteristics of 41 patients who completed the questionnaire are shown in Table 1. Twenty-three (56.0%) patients were male and 17 (41%) patients were over 65 years old. The first questionnaire was answered after a median time on PD of 296.5 (24.5–829.5) days. In the 20 patients who had 2 evaluations, the median (IQR) time elapsed between assessments was 205 (460–539) days.

For the initial 41 patients 9 were transferred to HD for noncompliance in 4, poor peritoneal clearances in 2, peritonitis in 2, and 1 hernia. Eleven patients were transplanted and 1 died.

Of the 20 patients censored in the second survey by the end of our analysis 10 were still on PD, 2 were transferred to HD, 6 had a renal transplant, 1 recovered renal function, and 1 died.

The median number of symptoms per patient was 10.0 (7.0–12.5) and 8.5 (6.2–13.0) for the first and second assessments, respectively (*P*-0.845).Table 2 shows scores obtained for both questionnaires in the 20 patients who repeated the survey. No statistical difference was disclosed between total scores and abdominal scores between the two evaluations.

The average number of symptoms per patient was 9.4 ± 4.2 and 9.6 ± 3.8 in the first and second assessments, respectively. Only one patient had no symptoms in the first survey, while 2 patients had 16 symptoms. The most prevalent symptoms in both surveys were lack of energy, itching, cramps, poor sleep, and shortness of in 36, 32, 30, 28, and 24 patients, respectively. Regarding the abdominal symptoms the most prevalent was loss of appetite followed by constipation and abdominal distention present in 26, 21, and 20 patients, respectively.

The only symptom that decreased the second survey was cramps, with an initial score of 1.8, and a score of 1.1 in the second evaluation, although the difference was not statistically significant (*P* = 0.120). 

There were no significant differences between the initial and subsequent evaluation of the total scores in subgroup analysis: diabetics versus nondiabetics (*P* = 0.771), age above or below 65 years (*P* = 0.669), male versus female (*P* = 0.096), and modality of PD (*P* = 0.659). 

## 4. Discussion

The pathophysiology of underlying symptoms in renal patients, especially in peritoneal dialysis, is still unclear. Most studies evaluating symptoms have been performed in haemodialysis patients. Our demographic data is similar to other studies with end-stage kidney disease patients, with a higher prevalence of the male gender and an increased number of diabetics [11–14].

This study shows that specific abdominal symptoms are few, the loss of appetite and constipation could be easily related to disease itself. The one related to the therapy was abdominal distention, but physical symptoms are more frequent in peritoneal dialysis patient, as is in hemodialysis patients. Although we used a different instrument, the number of symptoms seems to be similar to the report by Danquah and coworkers [5] with an average number of symptoms in the first and second HD session of the week was 9.77 and 7.51, respectively. Davison [12] has also found an elevated number of symptoms in PD and HD patient.

Fatigue or weakness is a highly prevalent complaint experienced by people with chronic illness. However it is a nonspecific one that can be conceptualized as located on a continuum between tiredness and exhaustion at one end and with vitality—being full of life and energy—at the opposite end [15]. Fatigue may also be characterized as lack of energy, and is reported in the majority of studies as the most prevalent symptom in end stage kidney disease population, experienced at some degree in 50–70% of dialysis patients [5, 15–17]. In our study, looking at PD patients, it was present in 88% of patients.

Muscle cramps is a well known distressing adverse effect that may arise during a hemodialysis session, much related to the volume of ultrafiltration needed, that can reach an occurrence of 10−20% [18–20]. Surprisingly our prevalence of muscle cramps was far higher than in hemodialysis patients. In a study by Weisbord et al. [17] the prevalence was of 43% in a cohort of both hemodialysis and peritoneal dialysis patients. Nonetheless occurrence of cramps in our study decreased over time perhaps due to a better understanding of volume management by the patients.

Two patients with the same clinical status and dialysis prescription can have different perception of their illness and manifest diverse symptoms. Symptoms are a subjective manifestation resulting from the interactions between the patient's overall conditions, emotions, background, and disease and the way in which they are perceived. Therefore it is not surprising that dialysis patient's perception may be more important than objective clinical assessments [10].

The weak association between symptoms and clinical variables is in line with other studies on dialysis patients [13]. It may be hard for the clinician to understand a patient's reported symptoms, since they are poorly related to any objective clinical indicator of uremia. This implies that clinical interventions aimed at reducing uraemia and improving quality of life might not necessarily correlates meaningfully to patients' subjective perception [10]. 

The sample size in the present study is a limitation of the present study, as are the patients being at a single unit, and the use of an author designed questionnaire. Despite such weakness we believe that this is a relevant study; it is the only study assessing symptoms specifically in patients on PD. 

## 5. Conclusion

This study demonstrates that physical symptoms are frequent and troublesome and remain stable throughout time, despite dialysis treatment. The most frequent abdominal symptom related to the therapy was abdominal distention. Symptoms reported are predominantly nonspecific. Further investigation is needed to establish whether symptom scores have a predictive value on dialysis outcomes.

## Figures and Tables

**Table 1:** Baseline demographic and clinical characteristics (41 patients).

Variable	Summary
Gender—male, *n* (%)	23 (56.0)
Diabetes mellitus, *n* (%)	8 (19.5%)
Type of PD modality, *n* (%)	
APD	17 (41.5)
CAPD	21 (51.5)
Total number of symptoms	
Median (IQR)	10.0 (7–12.5)
Total scores	
Median (IQR)	28.0 (18.5–36.0)
Abdominal score	
Median (IQR)	6.0 (2.5–9.5)

IQR: interquartile range, HD: hemodialysis, PD: peritoneal dialysis, APD: automated peritoneal dialysis, HD: hemodialysis.

**Table 2:** Total and abdominal symptoms score in patients that repeated the survey (*n* = 20).

Variable	Questionnaires		*P*
	1°	2°
Total score			
Median (IQR)	30.5 (19.5–36.0)	30.0 (17.7–37.3)	0.720
Min–max	10–54	10–50	
Abdominal score			
Median (IQR)	5.5 (5.0–11.2)	5.5 (3.2–9.0)	0.860
Min–max	0–20	0–16	

IQR: interquartile range.
